# Retaining Ligaments of the Face: Still Important in Modern Approach in Mid-Face and Neck Lift?

**DOI:** 10.3390/jpm15120582

**Published:** 2025-12-01

**Authors:** Mauro Tarallo, Matteo Cilluffo, Francesco Papa, Benedetta Fanelli

**Affiliations:** Department of Surgery “P. Valdoni”, Sapienza University of Rome, 00185 Rome, Italy; mauro.tarallo@uniroma1.it (M.T.); francesco.papa@uniroma1.it (F.P.); b.fanelli@ausl.latina.it (B.F.)

**Keywords:** retaining ligaments, preservation, releasing

## Abstract

**Background:** Facial retaining ligaments are pivotal in maintaining facial structure and are increasingly recognized as critical components in modern facelift procedures. Their age-related laxity contributes to facial sagging, jowling, and volume descent, necessitating a detailed understanding of their anatomy and function to achieve natural and lasting aesthetic outcomes. Despite advances in technique, there remains an ongoing debate regarding whether surgical preservation or release of these ligaments yields superior results. **Methods:** This narrative review analyzes peer-reviewed literature on various facelift techniques, focusing specifically on how each approach manages retaining ligaments. Techniques assessed include subcutaneous, SMAS, deep plane, composite, subperiosteal, and extended SMAS rhytidectomies, as well as more recent methods such as the MACS lift and PRESTO facelift. Anatomical variations and their surgical implications were evaluated, alongside aesthetic outcomes, recovery profiles, and complication risks. **Results:** Ligament-releasing techniques, such as the deep plane and extended SMAS facelifts, allow for greater tissue mobilization, improved repositioning of midfacial and cervical tissues. Conversely, ligament-preserving techniques, such as the MACS and PRESTO lifts, offer safer, less invasive, though with more limited correction in severe laxity. The review emphasizes that variability in ligament anatomy requires a patient-specific surgical plan to optimize results. **Conclusions:** The management of retaining ligaments remains a cornerstone of facial rejuvenation strategies. Surgical success hinges on a tailored approach, balancing the need for comprehensive lift with the preservation of facial identity and anatomical safety. Further clinical research and advancements in imaging and surgical technology are needed to refine technique selection and enhance long-term outcomes.

## 1. Introduction

The term “retaining ligament” was first introduced by Dr. Mar McGregor to describe specific areas of fibrous attachment within the face [1]. Since then, numerous anatomical studies have been conducted, shedding light on similar structures, identified with different names such as “patch,” “ligament,” “fascia,” or “septum” [2]. Unfortunately, this use of such a nomenclature has often led to confusion regarding the precise description of facial retaining ligaments. While there is general agreement on the terminology for key structures like the zygomatic and masseteric ligaments, discrepancies persist in defining the extent and function of subcutaneous ligamentous attachments [3].

Moreover, this understanding is crucial, especially in the case of procedures like mid-face and neck lifts, where the debate over the release or preservation of these ligaments continues to evolve. There is a growing appreciation for the role of these ligaments in maintaining facial structure and preventing sagging, yet ongoing discussions focus around whether complete release or careful preservation is essential for achieving optimal, natural, and long-lasting outcomes.

Some authors, delving further into the complexities of facial anatomy, advocate for a comprehensive release of retaining ligaments, arguing that it allows for maximal mobilization and repositioning of facial tissues. This would enable surgeons to lift and reshape the face more dramatically, enhancing the aesthetic outcome. On the other hand, others emphasize the importance of ligament preservation, noting that maintaining these structural connections helps preserve the patient’s unique facial phenotype, avoiding an over “pulled” or unnatural appearance.

Overall, these differing views highlight the broader implications for facial rejuvenation, where individualized approaches are crucial. Strengthening the argument for both techniques, the choice to release or preserve facial ligaments must be carefully tailored to each patient’s anatomy and aesthetic goals.

In 1989, Furnas [4] highlighted the importance of facial ligaments as anatomical structures that anchor skin and soft tissues to the facial skeleton. Key ligaments, such as the zygomatic, mandibular, and masseteric ligaments, are essential for facial contour and expression. Aging affects these ligaments, contributing to midface and neck sagging. Despite foundational studies by Furnas, alternative terminologies introduced by later researchers have complicated the classification of these ligaments [5,6].

Terminology inconsistencies are significant, with differing views on whether ligaments should be classified as “cutaneous” or based on their attachments and origin. For example, Mendelson [7] proposed a system distinguishing between adhesion, septum, and true ligaments, avoiding the term “cutaneous” altogether.

During surgery, traditional facelift techniques are often used to disrupt these ligaments through subcutaneous or deep-plane dissections. However, more contemporary approaches increasingly emphasize the preservation or selective release of these ligaments, depending on the desired outcome. Some surgeons view facial retaining ligaments as the “guardians of facial identity” [8], arguing that their preservation is essential for maintaining a patient’s natural facial features and individual phenotype, all while achieving a rejuvenated appearance.

On the other hand, proponents of complete ligament release argue that fully mobilizing facial tissues allows for a more comprehensive correction of age-related changes, thereby ensuring longer-lasting results [9]. In addition, this approach may offer greater flexibility in reshaping the facial contour. Therefore, the balance between these two approaches—preservation versus release—remains a topic of ongoing debate. More importantly, the goal is to achieve natural and individualized outcomes for patients undergoing facial rejuvenation procedures, further strengthening the need for tailored approaches based on individual anatomy and aesthetic goals.

## 2. Relevant Sections

### 2.1. Anatomical Overview of Retaining Ligaments

Facial retaining ligaments are connective tissue bands crucial for structural integrity and mobility. Their anatomy is variable, especially in the upper cheek, though key ligaments like the zygomatic and masseteric ligaments align consistently with landmarks such as the zygomaticus major muscle and facial nerve branches.

**Figure 1:**
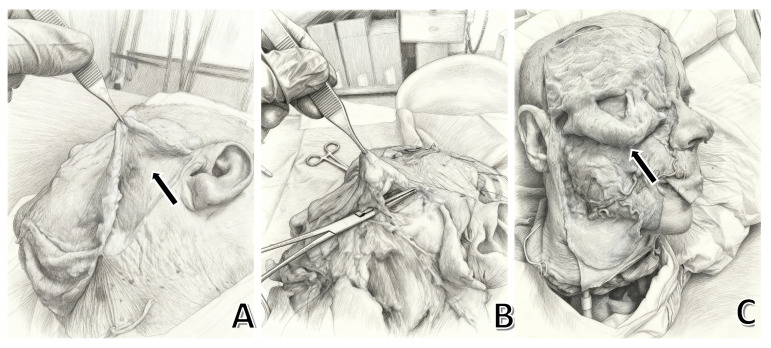
(**A**) Elevation of the SMAS flap during a cadaveric dissection. In the deep plane, the parotidomasseteric fascia is visible. The arrow indicates the lateral portion of the zygomatic (or McGregor’s) retaining ligament. (**B**) As the dissection progresses medially, the ligament is progressively isolated and becomes clearly identifiable (**C**). After extensive dissection, the medial portion of the ligament can also be appreciated (black arrow).

**Figure 2:**
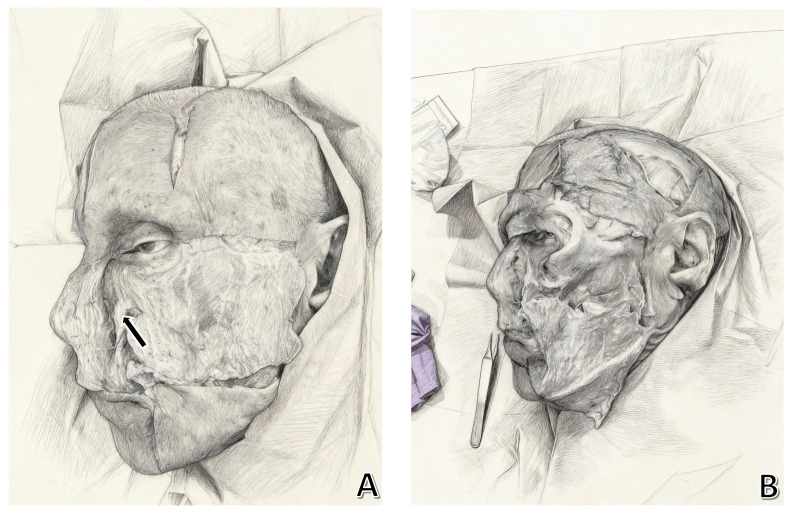
(**A**) After subcutaneous dissection, the SMAS is exposed with visualization of the orbicularis retaining ligaments (arrow). (**B**) Removal of SMAS, its cranial continuation as the superficial temporal fascia and its caudal continuation as the SMAS-platysma complex can be identified.

Key structures include:

- McGregor’s patch [1]: A fibrous attachment linked to the parotid fascia and cheek dermis, associated with critical landmarks like the parotid duct and transverse facial artery (Figure 1).

- Platysma auricular ligament (PAL) [4]: Described by Furnas, this ligament anchors the platysma to the skin. Later studies revised its connection to the parotid fascia.
- Tear trough–orbicularis retaining ligament complex (Figure 2) [10]: Crucial for midcheek stability, its release is vital in surgeries to elevate facial tissues and achieve better results.

**Figure 3:**
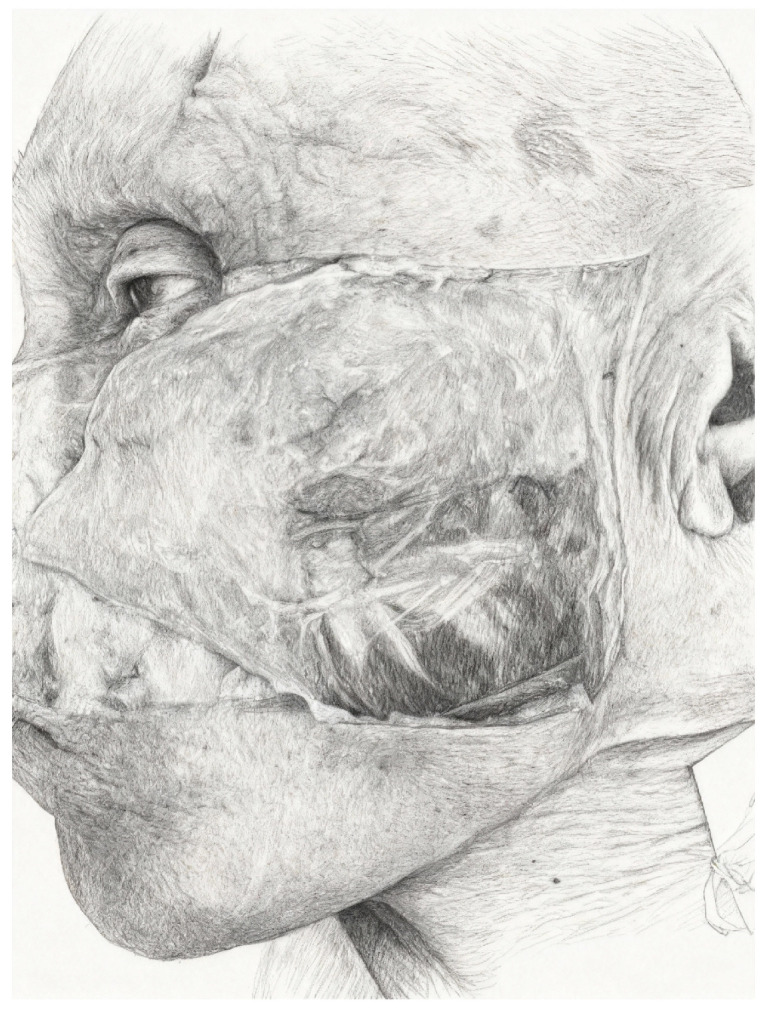
Elevated SMAS flap: the deep layer, corresponding to the parotidomasseteric fascia, is exposed, with the branches of the facial nerve and the transverse facial artery visible in transparency.

The premaxillary and prezygomatic spaces [11] are pivotal for independent movement of facial regions. These spaces house essential nerves, arteries, and fat pads, playing critical roles in both anatomy and surgery (Figure 3).

Other crucial ligaments are listed in Table 1.

Undoubtedly, the number, density, and configuration of the retaining ligaments in the upper cheek exhibit significant variability among individuals. Nonetheless, the position of the main zygomatic and upper masseteric ligaments remains consistently aligned with the zygomaticus major muscle and the zygomatic branches. This consistency applies both to their topographic anatomy, spanning from lateral to medial, and, more importantly, to their depth, extending from superficial to deep layers. Hence, understanding these nuances and the inherent variability in the complex three-dimensional anatomy of the midface during sub-SMAS dissection can enhance the safety and precision of facial surgeries. Moreover, this awareness ensures that surgeons can tailor their approach, further improving the outcomes of midface rejuvenation procedures. Surgical techniques involving these ligaments focus on balancing release and preservation to ensure safety, optimal aesthetic results, and individualized approaches. Variations in ligament configuration and anatomy require precise understanding to improve outcomes, especially in midface rejuvenation and avoiding complications.

### 2.2. The Role of Retaining Ligaments in Aging

Aging impacts retaining ligaments by causing them to weaken and stretch, leading to facial fat pad descent, increased skin laxity, jowls, deepening nasolabial folds, and platysmal banding [12]. These changes, combined with soft tissue deflation and reduced elasticity, contribute to facial aging [13]. The exact role of ligaments in this process remains debated, with some attributing aging to ligament laxity, while others suggest it stems from unsupported soft tissue descent.

Key grooves, like the nasojugal and palpebromalar grooves, highlight ligament-related changes, such as the tear trough and orbicularis retaining ligaments at the lid-cheek junction. Zygomatic ligaments contribute to malar bags and festoons [14], while the mandibular ligament influences jowl formation. Aging also affects spaces like the prezygomatic and premasseteric spaces, which sag and alter facial contours.

Modern facial rejuvenation techniques emphasize ligament release and tissue redraping to restore natural contours. Releasing ligaments reduces tension and avoids unnatural tightness. Different surgical planes—subcutaneous, sub-SMAS, and subperiosteal—determine ligament handling, with care taken to protect nerves like the facial and marginal mandibular nerves. Specific techniques, such as brow lifts, midface lifts, and blepharoplasty, rely on ligament release to reposition tissues and improve outcomes.

Despite their variability, retaining ligaments like the zygomatic and mandibular ligaments must be managed carefully during surgery. Their anatomy, density, and location differ among individuals, requiring tailored approaches. Advances in understanding and manipulating these structures have significantly improved natural and long-lasting results in facial surgery.

## 3. Surgical Techniques

### 3.1. Subcutaneous Facelift

In the traditional subcutaneous facelift, widely performed before the 1970s [15], surgeons elevated only a superficial skin flap without delving into the deeper structures. At present, this technique is considered more limited, as it relies heavily on skin tension for closure. The subcutaneous facelift involves dissection within the superficial plane, separating the skin from underlying structures without addressing the SMAS (Superficial Musculoaponeurotic System). While this approach offers reduced invasiveness and quicker recovery times, it is associated with less durable outcomes due to its limited impact on deeper structures.

### 3.2. SMAS Rhytidectomy

Ever since the introduction of the superficial muscular aponeurotic system (SMAS) rhytidectomy described by Skoog [16] in the 1970s, a more advanced two-layer approach has emerged. This method raises both the skin and the SMAS layer, a deeper tissue structure that bears more tension. While retaining ligaments remained largely preserved, manipulating the SMAS allowed for more effective facial lifting without placing undue tension on the skin, thereby improving both aesthetic outcomes and the longevity of the results [17].

### 3.3. Minilift

A less invasive option, the mini-lift [18] primarily focuses on the superficial layers of the face, involving minimal or no manipulation of retaining ligaments. This technique, described by Baker and Stephenson in the early ’70s [19,20], is ideal for patients with mild to moderate facial laxity and offers a shorter recovery period. However, the limited scope of ligament release often results in less dramatic and shorter-lasting improvements compared to more extensive techniques.

### 3.4. Subperiosteal Facelift

The subperiosteal approach [21], presented by Tessier in 1986, involves dissection beneath the periosteum, lifting both periosteal and ligamentous structures from their bony anchors. Retaining ligaments, such as the zygomatic and orbicularis ligaments, are released along with the periosteum to enable significant tissue mobilization. This technique is particularly effective in addressing mid-face laxity and infraorbital contour irregularities. However, the extensive nature of the dissection increases recovery times and demands meticulous surgical execution.

### 3.5. Retaining Ligaments and Advanced Facelift Techniques

The development of deep plane rhytidectomy by Hamra [22] marked a significant milestone in addressing the limitations of earlier techniques, particularly regarding nasolabial fold laxity. Dissection occurs beneath the SMAS, and key retaining ligaments, including the zygomatic, masseteric, and mandibular ligaments, are released to allow for broader mobilization of facial tissues. This comprehensive ligament release is particularly effective in repositioning the mid-face and addressing nasolabial folds. The procedure necessitates advanced surgical skills and careful handling to minimize complications, such as nerve injuries.

On the other hand, Owsley’s bidirectional technique [23], which elevates the SMAS and skin in different directions without releasing the facial retaining ligaments, offers limited vertical lift. While it provides some improvement in areas such as the neck and jawline, it does not address the ligament tethering in the midface and upper face.

### 3.6. Ligament Preservation in Vertical Lifts

While the release of retaining ligaments is crucial for achieving more extensive lifts, some modern techniques, such as the minimal access cranial suspension (MACS) lift [24], described by Tonnard and Verpaele in 2007, focus on preserving these structures. The MACS lift, which emphasizes a vertical vector of lift, typically avoids dissecting facial ligaments, relying instead on sutures to suspend the SMAS. However, it may require additional procedures for comprehensive neck correction, given the limitations of ligament preservation.

### 3.7. Extended Deep Plane Facelift

The extended deep plane rhytidectomy, as advocated by Jacono (2011) and others [25], takes the concept of ligament release even further by incorporating a more aggressive release of multiple facial retaining ligaments, including the zygomatic, mandibular, masseteric and neck ligaments. By fully mobilizing the SMAS-platysma complex and separating it from its ligamentous attachments, this technique enables more dramatic vertical and lateral repositioning of facial tissues [26]. In addition, releasing the cervical retaining ligaments significantly improves neck contour and submental definition, addressing issues like submandibular gland ptosis, which are challenging to correct with less invasive approaches. While effective for patients with pronounced laxity, the extended SMAS facelift requires significant surgical precision to avoid complications, such as buccal fat pad herniation or nerve damage [27].

### 3.8. Vertical Facelift

The vertical neck lift, refined by Jacono and Talei in 2014 [28], combines vertical lifting with ligament release. In this technique, the SMAS-platysma complex is elevated as a unit and draped vertically after releasing the zygomatic, mandibular, and cervical retaining ligaments. Hence, this technique allows for more complete mobilization of facial and neck tissues. The vertical vector facelift emphasizes the repositioning of facial tissues in a direction countering gravitational forces.

### 3.9. PRESTO-Lift

Described by Funk in 2017, the PRESTO (preservation of retaining ligaments and SMAS tethering) facelift technique [8] emphasizes the preservation of retaining ligaments and SMAS intersegmental connections, maintaining the patient’s individual facial phenotype. Unlike traditional approaches that often sever retaining ligaments, this technique carefully distends most ligaments, except the zygomatic-cutaneous ligament, when necessary, to retain natural points of tissue fixation. The preservation of SMAS connections and the zygomatic SMAS border further supports the structural integrity of facial compartments. By avoiding excessive disruption, the PRESTO facelift addresses age-related changes, such as thinning and lengthening of the lower eyelid, through midfacial-submalar preparation and optimizes neck contour with subplatysmal disconnection and modeling. The direction of SMAS traction allows for a harmonious transition between the zygomatic bone and the lateral orbital region and ensures a lift of the malar fat pad with a volumizing effect. This combination of techniques avoids integumental over-extension by preserving the individual, dynamic, and anatomical boundaries, while maintaining excellent mobility [29].

## 4. Discussion

This review synthesized peer-reviewed literature addressing facelifts, with particular attention to the management of retaining ligaments—a critical yet debated element in modern rhytidectomy. Studies were included according to whether they advocated release or preservation of these ligaments, thereby allowing comparison of divergent philosophies and the reasoning underpinning each approach. Beyond technical nuance, this issue reflects broader questions of surgical philosophy: whether optimal rejuvenation is best achieved through extensive mobilization and vector control, or through selective preservation that safeguards vascularity and minimizes morbidity (Table 2). The analysis also considered postoperative recovery, complication rates, and the long-term stability of results, thereby providing a perspective on how ligament management influences decision-making in facial rejuvenation surgery.

The evolution of facelift surgery provides essential context for understanding the centrality of this debate [30]. Early techniques focused on skin-only excision and redraping, which often yielded short-lived results and conspicuous stigmata due to lateral tension. The subsequent introduction of SMAS manipulation allowed surgeons to target deeper support structures, extending the longevity and naturalness of outcomes [31]. More recently, deep-plane and composite facelifts have emphasized vertical and superolateral vector repositioning, directly addressing midface descent, jowling, and cervical laxity while reducing reliance on skin tension [32]. Refinements in dissection planes, suture fixation, and atraumatic handling of tissues have expanded the safety profile of these procedures, lowering the rates of contour irregularities, alopecia, and nerve injury [33]. This historical progression underscores a broader paradigm shift: from skin manipulation to anatomic restoration, with ligament management representing the next frontier of debate.

Retaining ligaments—including the zygomatic, masseteric, mandibular, and orbicularis retaining ligaments—play a defining role in facial architecture. These structures tether soft tissues to underlying bone and fascia, thereby maintaining youthful contours and supporting dynamic facial expression. Their surgical treatment profoundly influences the degree of mobilization that can be achieved, the vector of redraping, and the overall aesthetic balance of the rejuvenated face [34]. High-resolution cadaveric and imaging studies have elucidated their morphology and interindividual variability, clarifying the relevance of glide planes such as the prezygomatic and premasseteric spaces. Release of these ligaments, particularly the orbicularis retaining ligament (ORL), facilitates en bloc mobilization of midface tissues, thereby counteracting lid–cheek elongation and malar mound formation [35]. Conversely, their preservation can maintain vascularity and structural stability but necessarily limits the extent of vector repositioning.

Clinical evidence increasingly informs this anatomical debate. A recent meta-analysis encompassing nearly 11,000 cases demonstrated comparable safety between deep-plane and SMAS approaches, with hematoma rates of approximately 3% versus 2%, and nerve injuries largely transient in both cohorts [36]. Importantly, deep-plane techniques provided superior midface elevation and durability, challenging the long-standing assumption that extensive release inevitably carries higher risk. These findings suggest that procedural selection should prioritize anatomical indication and patient-specific goals rather than be constrained by risk-averse bias.

Among the techniques that epitomize controlled ligament release, the Finger-Assisted Malar Elevation (FAME) method warrants special mention. By employing tactile dissection through the prezygomatic space, surgeons can selectively divide the zygomatic cutaneous ligaments under direct control, thereby minimizing the risk of injury to facial nerve subbranches and preserving the malar fat pad en bloc [37,38]. When incorporated into composite-flap rhytidectomy, this maneuver enables balanced midface repositioning without the need for an additional midface lift, illustrating how anatomy-guided strategies can maximize efficiency, safety, and aesthetic harmony. The FAME technique exemplifies a broader principle: precise, anatomy-based release of select ligaments can enhance results while avoiding unnecessary disruption of supportive structures.

Preservation-oriented strategies, such as the Preservation Face Lift and rotating-pedicle variants, represent the opposite end of the spectrum. These methods minimize dissection, maintain vascular and ligamentous attachments, and reduce operative morbidity. Reported benefits include shortened drainage times, decreased risk of flap ischemia, and more rapid recovery, making them especially suitable for younger patients or those seeking conservative enhancement [39,40]. Aesthetic advantages include well-defined jawline refinement and maintenance of soft tissue vascularity. However, the very tethers that are preserved restrict vertical and lateral repositioning, limiting the magnitude of correction in patients with advanced ptosis, thick tissues, or significant cervical laxity.

These contrasting philosophies should not be perceived as mutually exclusive but rather as points along a continuum of surgical strategies. Release-forward approaches confer greater mobilization, vector control, and long-term stability, while preservation-oriented methods offer lower morbidity, vascular safety, and faster recovery. The discerning surgeon’s responsibility lies in tailoring the operative plan to the individual—accounting for facial anatomy, degree of ptosis, soft tissue volume, prior surgeries, vascular status, and patient expectations regarding downtime versus durability. In this sense, ligament management encapsulates the broader ethos of modern facial rejuvenation: personalization of technique to achieve balance between safety, efficacy, and natural outcomes.

The integration of adjunctive and nonsurgical interventions further reinforces this multimodal philosophy. Contemporary facelifting is rarely performed in isolation; rather, it is embedded within a comprehensive rejuvenation strategy. Ancillary procedures such as brow lifting, lip lift, and buccal fat reduction restore periorbital and perioral balance, complementing midface and cervical elevation [41]. Energy-based devices—including radiofrequency, ultrasound, microneedling, and lasers—are increasingly employed to enhance skin quality and texture, while traditional methods such as dermabrasion and chemical peeling remain valuable for addressing pigmentation and fine rhytids [42,43]. Injectable therapies, including neuromodulators, volumizing fillers, and biostimulatory agents such as platelet-rich plasma, platelet-rich fibrin, poly-L-lactic acid, and calcium hydroxylapatite, provide volumetric and biologic enhancements [44]. Finally, perioperative cosmeceuticals further optimize skin health and healing [38,45]. Collectively, these adjuncts illustrate the multifactorial nature of modern rejuvenation, where surgical repositioning is integrated with regenerative and resurfacing modalities to produce comprehensive and natural outcomes.

Safety refinements remain a central priority. Hematoma is consistently reported as the most frequent complication of facelift surgery, with rates ranging between 1% and 14% [46]. Established risk factors include perioperative hypertension, male sex, and anticoagulant use. Preventive strategies emphasize meticulous intraoperative hemostasis, compression dressings, and strict perioperative blood pressure management—often aiming for systolic values below 120 mmHg [47]. Traditional drains have not consistently demonstrated efficacy in reducing hematoma rates [48], prompting many surgeons to adopt alternatives such as fibrin sealants and hemostatic nets, the latter minimizing dead space and shear forces in the subcutaneous plane. Quilting sutures represent another adjunct, though they carry the potential drawback of pigmentation changes, which may be mitigated by finer calibers [49].

Pharmacologic innovations also contribute to enhanced safety. Tranexamic acid (TXA), administered either intravenously or by local infiltration, has been investigated for its capacity to reduce intraoperative bleeding, postoperative edema, and ecchymosis. Randomized controlled trials suggest that intravenous TXA provides particular benefit in reducing bruising and serosanguinous collections [50]. However, concerns have been raised regarding flap necrosis and ischemia when TXA is applied locally in high concentrations, with systematic reviews attributing most reported cases of necrosis to this route of delivery [51]. Accordingly, careful selection of administration route and dosage is crucial to maximize benefit while minimizing risk.

Finally, anesthetic approaches are evolving in parallel with surgical refinements. Increasing numbers of facelifts are now performed under local anesthesia alone, driven both by patient demand for reduced anesthetic risk and by technical advances that permit comfortable wide-awake surgery [52]. Large case series indicate that this approach not only eliminates risks associated with general anesthesia but may also reduce hematoma incidence, likely through more stable intraoperative hemodynamic control [53]. Furthermore, wide-awake protocols allow surgeons to combine facelifting with ancillary procedures in a single session, thereby improving efficiency and patient satisfaction. Nonetheless, anesthetic planning must remain individualized, balancing patient comfort, procedural complexity, and the need for perioperative safety.

This table aims to provide greater clarity regarding the indications for each facelift technique, highlighting their respective advantages and limitations. By comparing the different approaches—ranging from less invasive options such as the subcutaneous facelift or mini-lift, to more advanced procedures like the deep plane or extended deep plane facelift—the review offers guidance on selecting the most appropriate technique for each patient. In this way, surgeons can better tailor the surgical plan according to the degree of ptosis, patient age, anatomical features, and aesthetic expectations, ultimately optimizing outcomes.

The more invasive and ligament-releasing techniques (deep plane, extended deep plane, vertical lift) require advanced expertise: the learning curve is long, and technical errors increase the risk of complications such as nerve injuries, hematomas, or contour irregularities. Conversely, more conservative and preservation-oriented techniques (SMAS, MACS, PRESTO) have shorter learning curves and fewer complications, though they may deliver less dramatic and durable results in patients with marked aging. The literature emphasizes that outcomes and complication rates are strongly influenced by the surgeon’s experience, rather than the technique itself (Table 3).

## 5. Resource Implications

Beyond anatomical considerations and complication rates, practical aspects such as operating time, anesthesia requirements, recovery burden, and resource utilization significantly influence both patient counseling and surgical decision-making. Reported operative times vary considerably across techniques: less invasive procedures such as the mini-lift or MACS lift are often completed within 1–2 h, frequently under local anesthesia with or without sedation, while more extensive approaches (deep plane, extended deep plane, vertical lift, and subperiosteal rhytidectomy) may require 3–5 h under general anesthesia. Recovery profiles follow a similar gradient: limited dissection techniques allow for faster resolution of edema and ecchymosis, with many patients resuming normal activities within 7–10 days, whereas extended deep-plane and subperiosteal approaches are associated with longer downtime, often exceeding 2–3 weeks.

Resource utilization also differs: drains are less frequently required in short-scar or limited undermining procedures but remain common in extended dissections to reduce seroma or hematoma risk. The need for perioperative blood pressure control measures, compression dressings, and occasionally hemostatic adjuncts (e.g., fibrin sealants or hemostatic nets) is more pronounced in complex lifts. These differences underscore that surgical planning should weigh not only the anatomical and aesthetic goals but also the patient’s tolerance for anesthesia, expected recovery burden, and occupational or social demands regarding return-to-work intervals.

## 6. Limitations

This study has several important limitations. First, it is a narrative review rather than a systematic review. As such, no formal protocol for study selection, data extraction, or risk of bias assessment was applied. The body of evidence summarized here is, therefore, subject to selection bias, as included articles were chosen based on relevance and representativeness rather than through a comprehensive systematic search strategy. Second, the conclusions drawn reflect a synthesis of findings from heterogeneous studies, many of which vary in design, quality, follow-up duration, and reporting of outcomes. In several instances, statements about relative advantages or drawbacks of specific facelift techniques inevitably rely on subjective interpretation and comparative discussion across studies, rather than on direct head-to-head evidence. For this reason, the views expressed should be interpreted as a critical appraisal and expert perspective informed by the literature, rather than definitive evidence-based recommendations. Future systematic reviews and, ideally, randomized or prospective comparative studies are warranted to validate and strengthen the observations reported here.

## 7. Conclusions

There is a paucity of data comparing efficacy of rhytidectomy by technique. Studies on medium-term efficacy show that less invasive SMAS approaches have a greater recurrence of neck laxity than jowl reformation. However, the data remain too limited to draw conclusions, and the significance of this information is unclear.

Undoubtedly, the decision to release or preserve retaining ligaments during facelift surgery plays a central role in determining the extent of tissue mobilization and the overall effectiveness and longevity of the lift. Thus, the choice of technique depends on the individual patient’s anatomy, the extent of facial aging, and the desired aesthetic outcomes. There is a growing appreciation for the importance of tailoring these techniques to the patient’s needs to optimize results and minimize complications. The choice between releasing or preserving retaining ligaments depends on multiple factors, including the patient’s degree of facial aging, anatomical considerations, and desired aesthetic outcomes. Releasing the ligaments offers a more powerful lift with more comprehensive tissue mobilization, particularly in the lower face and neck. However, it requires technical precision and carries a greater risk of complications. On the other hand, preserving ligaments provides a safer, more conservative approach with natural results, though it may be less effective in addressing severe facial laxity. Ultimately, the decision to release or preserve these ligaments should be tailored to the individual patient, considering both aesthetic goals and the potential risks associated with each technique.

## 8. Future Directions

The retaining ligaments of the face continue to represent a cornerstone of modern facial rejuvenation strategies, particularly in mid-face and neck lift procedures. As surgical techniques evolve toward more customized, patient-centered approaches, the need for a deeper understanding of the anatomy, variability, and function of these structures becomes increasingly vital.

Future research should prioritize high-resolution imaging studies and cadaveric dissections to further elucidate the three-dimensional architecture and interindividual variability of retaining ligaments. This would allow for more precise preoperative planning and better identification of anatomical “danger zones” during surgical dissection, thereby minimizing complications and enhancing outcomes.

Moreover, comparative clinical studies assessing long-term aesthetic results, complication rates, and patient satisfaction across different ligament-preserving and ligament-releasing techniques are critically needed. Randomized controlled trials (RCTs) or large-scale, multicenter registries would help validate the efficacy and safety of various methods in diverse patient populations, including those with differing degrees of aging, ethnic backgrounds, and skin types.

Future research must aim to reconcile the persistent controversies in facelift anatomy and technique. Prospective, multicenter trials comparing preservation versus release strategies across phenotypically diverse cohorts are required, using standardized aesthetic outcomes and validated patient-reported measures such as the FACE-Q [54].

There is also potential for innovation in surgical instrumentation and technique, including the development of minimally invasive tools or robot-assisted systems designed to manipulate retaining ligaments with greater precision and reduced trauma. Adjunct technologies, such as intraoperative ultrasound or augmented reality-guided dissection, may soon enable surgeons to visualize ligamentous structures in real time, further refining surgical accuracy.

In addition, the integration of regenerative medicine—such as stem cell therapy, biostimulatory fillers, and scaffold materials—may offer promising adjuncts for reinforcing ligamentous structures non-surgically or enhancing tissue quality postoperatively.

## Figures and Tables

**Table 1:** Nomenclatures of retaining ligaments [3].

Cheek Ligaments	Synonym
MALAR AREA McGregor’s patch	Zygomatic ligament Zygomatic cutaneous ligament
PERI-AURICULAR AREA Platysma auricular ligament Parotid cutaneous ligament Platysma auricular fascia Temporoparotid fascia	Auricle-platysma ligament Platysma auricular fascia Preauricular parotid cutaneous ligament Platysma auricular ligament Parotid cutaneous ligament Platysma auricular fascia Auricle-platysma ligament Preauricular parotid cutaneous ligaments Lore’s fascia Lore’s fascia Tympanoparotid fascia
PERI-MASSETERIC AREA Anterior platysma-cutaneous ligament Platysma cutaneous ligament	Masseteric cutaneous ligament Parotidomasseteric cutaneous ligament Mandibular septum

**Table 2:** Comparative table of cervicofacial lifting techniques and management of retaining ligaments.

Technique	Dissection Plane	Ligament Management	Advantages	Limitations	Primary Indications
**Subcutaneous facelift**	Supra-SMAS	Generally preserved	Simple technique, quick recovery	Less durable results, limited effect	Mild laxity, young patients
**SMAS rhytidectomy**	Superficial SMAS	Preserved or plicated	Better support than cutaneous technique	Limited effect on midface	Mild-moderate ptosis, middle-aged patients
**Mini-lift**	Supra-SMAS	Not manipulated	Minimally invasive, short recovery time	Limited correction, shorter duration	Mild facial laxity
**Deep plane facelift**	Sub-SMAS	Released (zygomatic, masseteric, etc.)	Natural and long-lasting results in midface	Advanced technique, higher nerve risk	Moderate-severe ptosis of midface and neck
**Subperiosteal facelift**	Subperiosteal	Released with periosteum	Effective on mid-third, deep lifting	Longer recovery, complex technique	Midface ptosis, infraorbital deformities
**MACS-lift**	Supra-SMAS with suspension	Preserved	Minimally invasive, vertical suspension	Less neck correction	Mild-moderate ptosis, good skin tone
**Extended deep plane facelift**	Extended sub-SMAS	Extensively released	Complete 3D lift, effective on neck	Requires high surgical expertise	Advanced aging, severe ptosis
**Vertical lift**	Vertical sub-SMAS	Released (zygomatic, mandibular, cervical)	Anti-gravity lift, natural result	Very precise technique, increased complication risk	Advanced midface and neck ptosis
**PRESTO facelift**	Selective supra and sub-SMAS	Preserved (except selectively released)	Preserves phenotype, natural effect	Less vertical lift effect	Patients with subtle, refined features

**Table 3:** Comparison of technique by follow-up period, complications and learning curve.

Technique	Sample Size/Follow-Up	Complications	Operator Experience/Learning Curve
**Subcutaneous**	Historical data; limited follow-up	Hematoma, less durable results, early recurrence	Simple technique, short learning curve
**SMAS rhytidectomy**	Large series (>500 pts)	Hematoma ~2–3%, rare transient nerve injuries	Requires SMAS knowledge; moderate learning curve
**Mini-lift**	Small series; short follow-up	Low complication rates, shorter-lasting results	Simple, short learning curve
**Subperiosteal**	Specialized series; mid-long follow-up	Longer recovery, edema, higher nerve risk	Complex; long learning curve
**Deep plane**	Meta-analyses (~11,000 cases)	Hematoma ~3%, transient nerve injuries; durable outcomes	Advanced technique; long learning curve
**Extended deep plane**	Dedicated series; mid-long follow-up	Risk of buccal fat herniation, nerve damage	Very advanced, steep learning curve
**Vertical lift**	Specialized series	Similar to deep plane; risk of misplacement if inexperienced	High precision required; long curve
**MACS lift**	Medium-large series; intermediate follow-up	Low risk, rapid recovery; less neck correction	Simpler; short-medium curve
**PRESTO lift**	Initial series (Funk 2017) [8]	Minimal complications, natural results; less vertical effect	Newer approach; learning curve evolving

## Data Availability

No new data were created or analyzed in this study. Data sharing is not applicable to this article.
